# Unraveling Adrenal Oncocytoma: Clinical Presentation, Diagnosis, and Surgical Success

**DOI:** 10.7759/cureus.80891

**Published:** 2025-03-20

**Authors:** Arjun N, Dinesh Anne, Bhavya Shetty, Murali Velagapudi, Nirupam Nadella

**Affiliations:** 1 Department of Urology, Sapthagiri Institute of Medical Sciences and Research, Bangalore, IND; 2 Department of General Surgery, Dr. D. Y. Patil Medical College Hospital and Research Centre, Dr. D. Y. Patil Vidyapeeth (Deemed to be University), Pune, IND

**Keywords:** adrenal oncocytoma, adrenal tumor management, eosinophilic cytoplasm, laparoscopic adrenalectomy, oncocytic cells

## Abstract

Adrenal oncocytomas are rare benign tumors originating in the adrenal gland and feature eosinophilic, mitochondria-rich cytoplasm. These tumors are difficult to diagnose and manage since they develop infrequently and have nonspecific symptoms. We present the case of a 45-year-old male with no known comorbidities who came with complaints of nonspecific on-off abdominal pain and no other associated findings. Imaging indicated a 12 × 10 cm mass originating from the right adrenal gland, abutting the right kidney, with no involvement of major arteries. Biochemical studies suggested a non-functioning adrenal tumor with Adrenocortical Carcinoma and non-functioning Pheochromocytoma as differentials. The patient underwent a right laparoscopic adrenalectomy with the tumor not invading any other structures. The patient had an uneventful postoperative period. The histopathology exam revealed an adrenal mass with predominant oncocytoma features. The patient had an uneventful postoperative period. The diagnosis of adrenal oncocytoma has been established by histopathological analysis. This report underscores the significance of histological confirmation in adrenal tumors and the need for additional research to provide diagnostic and treatment guidelines for these uncommon neoplasms, given the limited number of reported cases.

## Introduction

Oncocytoma is a type of tumor that primarily affects the kidneys, salivary glands, thyroid, parathyroid, lacrimal gland, lungs, pancreas, and adrenal gland [1]. Only roughly 200 instances have been identified as adrenal origin thus far [2]. The kidney is the most typical site, accounting for 3%-5% of all renal tumors [3]. The initial account was published by Kakimoto et al. in 1986 [4]. Originally, adrenal oncocytoma (AOC) was thought to be a “silent” nonsecreting benign adrenal neoplasm. A few case reports have indicated that AOC might be hormonally active or malignant [5]. Thus, it seems that the natural history and the clinical behavior of these tumors are more complex than previously thought, and additional data are needed to elucidate their true significance [5]. AOC, although extremely rare in the adrenal cortex, should be included in the differential diagnosis of large, well-defined, and nonfunctioning adrenal tumors and should be managed appropriately [5]. Nevertheless, uncertainty still remains about the natural history and the true potential and significance of these neoplasms, and the mechanisms underlying oncocytic metaplasia are still obscure [5].

## Case presentation

A 45-year-old male patient, unique in his non-smoking status and absence of hypertension, presented to the outpatient department with complaints of non-specific on-off abdominal pain and dyslipidemia. The patient, an obese diabetic with no other comorbidity, had no palpable mass per abdomen. An ultrasound scan revealed a large, well-defined adrenal mass on the right side with an uncertain extension. The patient underwent a CT scan, which showed a 12 × 10 cm mass arising from the right adrenal gland abutting the right kidney with doubtful extension. Hormonal studies were done to rule out any functionality, with the urinary metanephrines, serum cortisol, dehydroepiandrosterone (DHEA), and overnight dexamethasone suppression test returning to normal. Before the operation, we suspected the case to be non-functional adrenocortical carcinoma.

**Figure 1:**
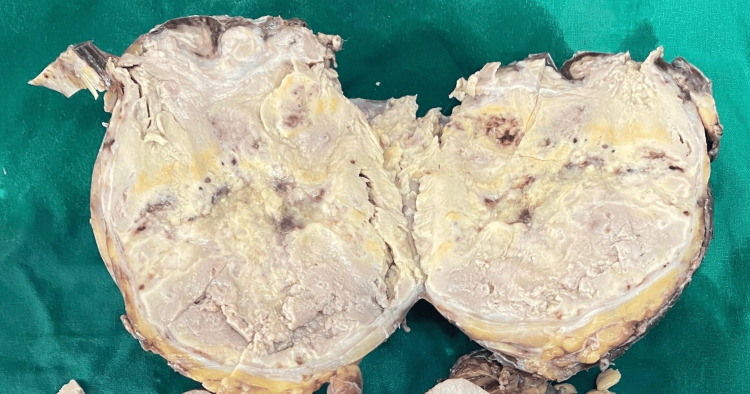
Gross pathology of adrenal oncocytoma: macroscopic characteristics Gross specimen of adrenal oncocytoma demonstrating a well-circumscribed, yellowish-brown tumor with a homogeneous cut surface and focal areas of hemorrhage.

The patient underwent an uneventful right laparoscopic assisted adrenalectomy, with the mass not seen extending into the kidney, and a 15 × 12 cm mass was excised (Figure 1) and subjected to histopathological examination. The postoperative course was uneventful, and the patient was discharged on the fourth postoperative day, showing promising signs of recovery.

**Figure 2:**
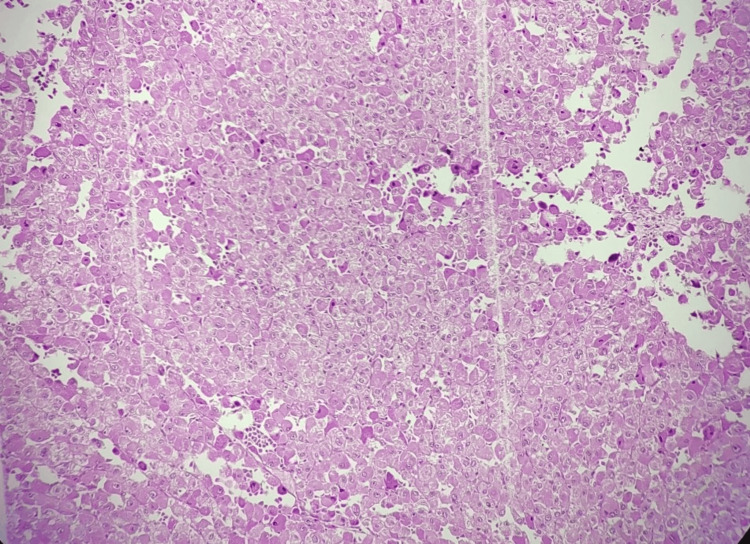
Histological features of adrenal oncocytoma showing oncocytic cell proliferation Hematoxylin and eosin-stained histological section of adrenal oncocytoma, demonstrating sheets of oncocytic cells with abundant granular, eosinophilic cytoplasm. The nuclei are uniform, round to oval, and show occasional prominent nucleoli. The section highlights areas with preserved tumor architecture and mild cytoplasmic granularity.

**Figure 3:**
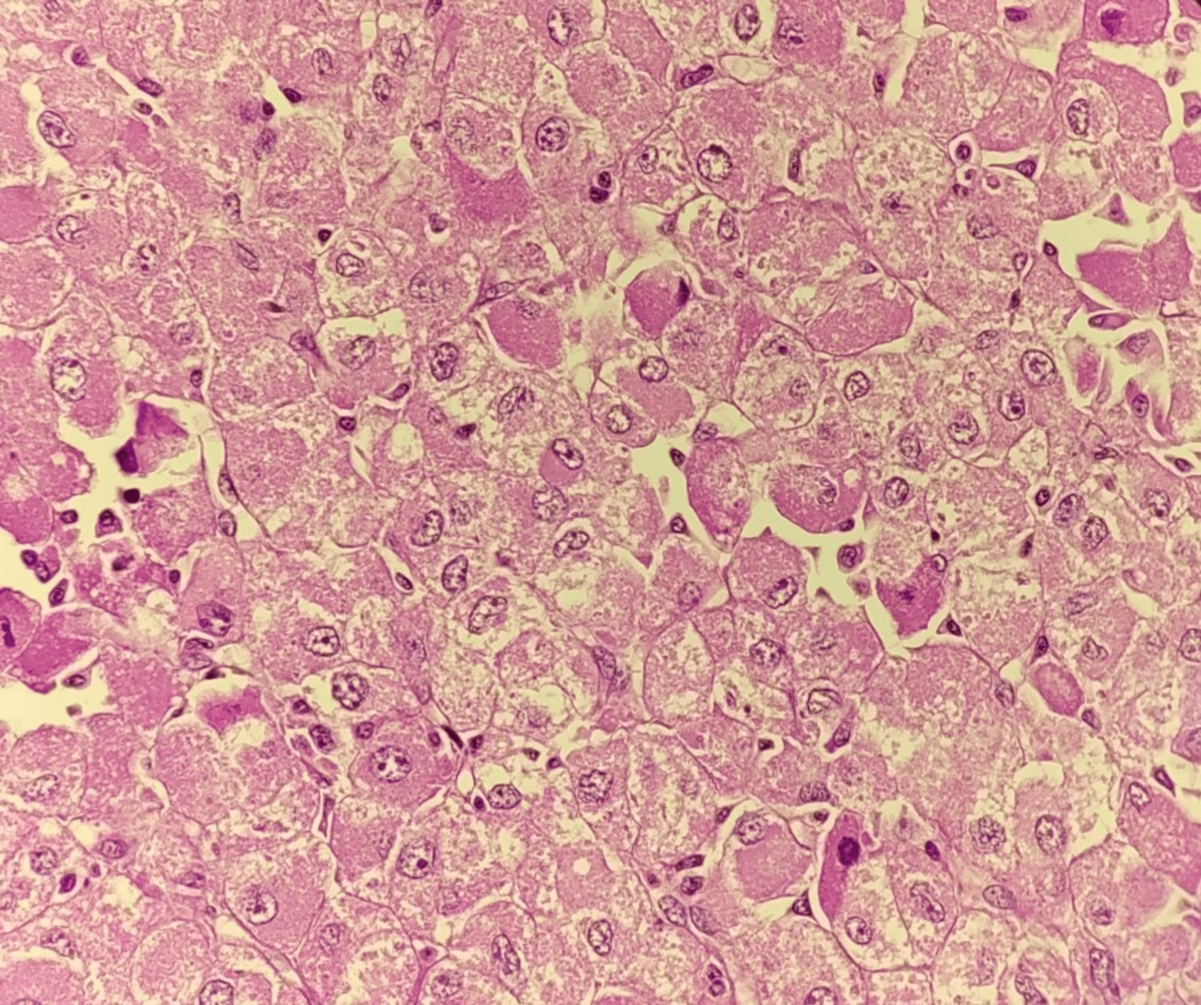
Histopathological features of adrenal oncocytoma: high-power view A hematoxylin and eosin-stained section of adrenal oncocytoma shows oncocytic cells with abundant eosinophilic, granular cytoplasm, and centrally located round nuclei.

**Figure 4:**
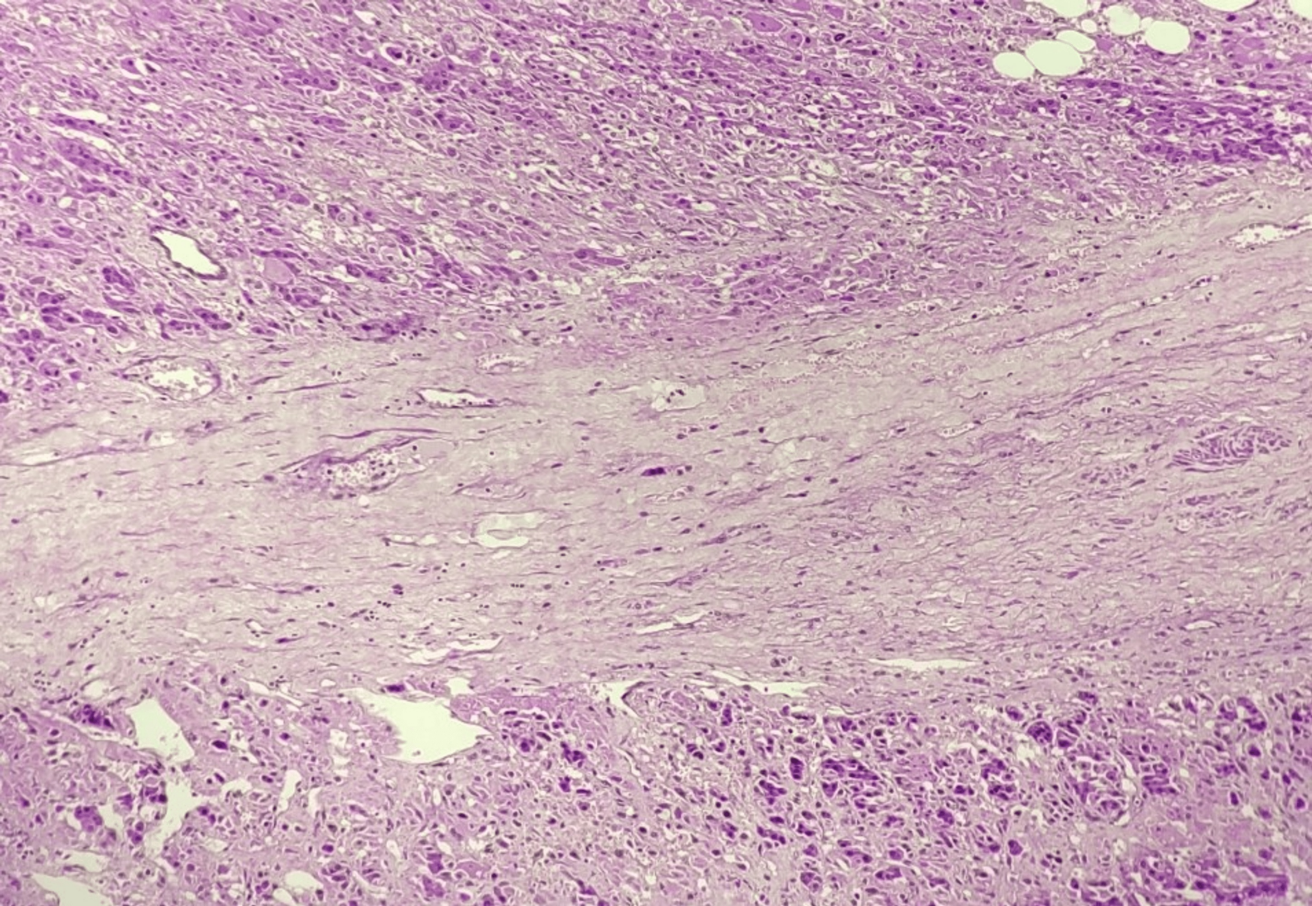
Histological features of adrenal oncocytoma with fibrous stromal component Hematoxylin and eosin-stained histological section of adrenal oncocytoma illustrating a fibrous stromal band traversing the tumor. The oncocytic cells, with abundant eosinophilic and granular cytoplasm, are seen at the periphery, while the central area consists of dense, collagenous stroma. Tumor cells are arranged in solid nests and cords with round to oval nuclei.

Histopathological study shows a tumor comprising variably sized nests, islands, sheets, and singly scattered tumor cells. Individual cells are predominantly oncocytic cells (>75%) having central to eccentrically placed round nuclei exhibiting a marked degree of nuclear atypia in the form of enlarged vesicular to hyperchromatic, pleomorphic nuclei, irregular nuclear contours, prominent nucleoli, atypical mitotic figures (6-8/50hpf) and abundant eosinophilic granular cytoplasm as shown in Figures 2-4. Also noted are a few intranuclear inclusions and many bizarre nuclei showing mitoses. Islands and nests of large cells with clear cytoplasm having pleomorphic nuclei are also seen (<25% of the tumor volume). Extensive areas of coagulative tumor necrosis (75%-80%) and hemorrhagic areas (10%-15%) are noted. The tumor is seen invading the adrenal thick fibrous capsule and periadrenal adipose tissue, suggesting an AOC. The patient was followed up postoperatively for six months and displayed no other complaints, with uneventful laboratory and imaging findings.

## Discussion

AOCs are rare benign adrenal gland tumors characterized by eosinophilic, mitochondria-rich cytoplasm [5]. Since their first description by Kakimoto et al. in 1986, fewer than 200 cases have been reported in the literature, making them an uncommon yet clinically significant entity [2,4]. The rarity of AOCs presents diagnostic and management challenges, mainly because they can be mistaken for other more common adrenal neoplasms, such as adrenocortical carcinoma and pheochromocytoma [6].

In our case, the patient presented with nonspecific on-off abdominal pain and underwent a series of imaging studies that revealed a large right adrenal mass measuring 12 × 10 cm. This presentation aligns with the typical clinical picture of AOCs, which often manifest as non-functional masses. Unlike other adrenal tumors that may cause hormonal syndromes, most oncocytomas are non-functional and present incidentally, complicating their detection [7]. The need for hormonal evaluation, even in seemingly non-functional cases, is essential for differentiating these tumors from functional adrenal lesions, as highlighted in previous literature [8,9].

When evaluating adrenal masses initially, imaging methods like CT and MRI are helpful [10]. However, imaging alone to differentiate oncocytomas from other adrenal tumors can be difficult because of their cellular makeup; AOCs frequently appear on radiographs as big, well-defined masses with heterogeneous enhancement [10]. These nonspecific imaging features highlight the need for histological evaluation for a conclusive diagnosis because it can cause misunderstanding with different neoplasms [11]. As is frequently observed with benign tumors, the CT scan of our patient revealed a mass next to the right kidney without evident invasion into other tissues [12]. Even though this imaging result suggested a benign lesion, the diagnosis needed to be confirmed by rigorous post-surgery histological examination.

The primary method for diagnosing AOC is still histopathology. The growth of oncocytes, which are big cells with plenty of mitochondria, defines this tumor [13]. When diagnosing oncocytomas, pathologists look for two distinguishing characteristics: the presence of a central nucleus and eosinophilic cytoplasm [13]. In our instance, the diagnosis based on imaging studies and clinical presentation was invalidated by the postoperative histological analysis, which verified the existence of oncocytoma characteristics. The surgical method is one of the most essential parts of treating AOCs. Because laparoscopic adrenalectomy is less intrusive, causes less discomfort after surgery, and has a shorter recovery period than open surgery, it has become the accepted treatment for adrenal tumors, including oncocytomas [14]. In line with data from other studies that indicate minimal complication rates and positive outcomes following laparoscopic surgeries, our patient had successful laparoscopic adrenalectomy and an uneventful postoperative course [14,15].

Although the course of AOCs is usually considered benign, it is impossible to rule out the possibility of malignant behavior [13]. Although the majority of instances progress benignly, a subset of these tumors has been known to display aggressive traits [13]. On a CT scan, benign oncocytic adrenocortical neoplasms can be differentiated from lipid-rich but not lipid-poor adenomas [16]. Large size, necrosis, and a reduced percentage of enhancement washout are characteristics that malignant ones share with adrenocortical carcinomas, making separation by CT extremely challenging [16]. Therefore, no CT or MRI criteria were available to distinguish between benign and malignant tumors [16]. Careful follow-up is thus crucial, especially in cases that debut with greater than typical sizes or abnormal histological characteristics [13]. There are a few particular recommendations for oncocytomas. However, subsequent research has suggested surgical resection [16-18]. As laparoscopic technology develops, laparoscopic surgery is growing in popularity, and laparoscopic surgery is becoming more and more widespread [17]. When the tumor is more significant than 6 cm, a laparotomy is advised to achieve a complete excision without tumor rupture [16]. For metastatic tumors, surgery is recommended if the metastasis is restricted and could be safely resected [19,20].

## Conclusions

Adrenocortical oncocytomas are tumors that can be benign or malignant with or without function. Surgical excision remains the primary treatment approach. Nonetheless, the results must be validated by large clinical samples, molecular research, precise diagnostic standards, and extended follow-up.
